# Supplementary material for: Alpha oscillations and event-related potentials reflect distinct dynamics of attribute construction and evidence accumulation in dietary decision making
Source: eLife. 2021 Jul 15;10:e60874. doi: 10.7554/eLife.60874 (PMC8318586; doi:10.7554/eLife.60874)
Supplement: Figure 2—source data 1. [file elife-60874-fig2-data1.docx]

|  | ***w_tastiness_*** | ***w_healthiness_*** | ***ValConst*** | ***trs*** | ***nondec*** | ***spbias*** |
| --- | --- | --- | --- | --- | --- | --- |
| **Natural** | .604 (std = .24) | .049 (std = .14) | -.57 (std = .46) | 1.89 (std = .26) | .370 (std=.13) | .478 (std=.20) |
| **Health** | .353 (std = .27) | .362 (std = .29) | -.469 (std = .46) | 2.00 (std = .26) | .372 (std=.13) | .47 (std=.20) |
| **Decrease** | .391 (std = .26) | .107 (std = .18) | -.833 (std = .57) | 2.16 (std = .39) | .337 (std=.15) | .469 (std=.23) |
