# Supplementary material for: Alpha oscillations and event-related potentials reflect distinct dynamics of attribute construction and evidence accumulation in dietary decision making
Source: eLife. 2021 Jul 15;10:e60874. doi: 10.7554/eLife.60874 (PMC8318586; doi:10.7554/eLife.60874)
Supplement: Transparent reporting form [file elife-60874-transrepform.pdf]

eLIFE
Westbrook Centre, Milton Road
Cambridge CB4 1YG
UK
P 01223 855340
W elifesciences.org
T @elife

eLife's transparent reporting form

We encourage authors to provide detailed information *within their submission* to facilitate the interpretation and replication of experiments. Authors can upload supporting documentation to indicate the use of appropriate reporting guidelines for health-related research (see EQUATOR Network), life science research (see the BioSharing Information Resource), or the ARRIVE guidelines for reporting work involving animal research. Where applicable, authors should refer to any relevant reporting standards documents in this form.

If you have any questions, please consult our Journal Policies and/or contact us: editorial@elifesciences.org.

Sample-size estimation

- You should state whether an appropriate sample size was computed when the study was being designed
- You should state the statistical method of sample size computation and any required assumptions
- If no explicit power analysis was used, you should describe how you decided what sample (replicate) size (number) to use

Please outline where this information can be found within the submission (e.g., sections or figure legends), or explain why this information doesn't apply to your submission:

We cite our pre-registration of this study (osf.io/ewtvx) on Page 19 of the manuscript, under *Methods/Subjects* which explains that a samples size of 50 subjects was decided based on previous research and our pre-registered analysis methods.

Our study was in part exploratory. No previous published research studied effects of different regulatory strategies on brain oscillations during value-based decision making, therefore we could not perform a precise power analysis. Instead, we decided our sample size by roughly adding 50% to the average of 28 usable subjects in Harris et al. (2013) and 23 subjects in Polania et al. (2014). These studies analyzed the event-related potentials during self-control and brain oscillations during value-based decision-making respectively, so we used a higher estimate of sample size to account for these differences.

Replicates

- You should report how often each experiment was performed
- You should include a definition of biological versus technical replication
- The data obtained should be provided and sufficient information should be provided to indicate the number of independent biological and/or technical replicates
- If you encountered any outliers, you should describe how these were handled
- Criteria for exclusion/inclusion of data should be clearly stated
- High-throughput sequence data should be uploaded before submission, with a private link for reviewers provided (these are available from both GEO and ArrayExpress)

Please outline where this information can be found within the submission (e.g., sections or figure legends), or explain why this information doesn't apply to your submission:

eLife Sciences Publications, Ltd is a limited liability non-profit non-stock corporation incorporated in the State of Delaware, USA, with company number 5030732, and is registered in the UK with company number FC030576 and branch number BR015634 at the address Westbrook Centre, Milton Road Cambridge CB4 1YG, UK | March 2019

eLIFE
Westbrook Centre, Milton Road
Cambridge CB4 1YG
UK
P 01223 855340
W elifesciences.org
T @elife

We describe the details of the paradigm on Pages 19-20 of the manuscript under *Methods/Task* including the number of blocks of trials that subjects performed in each condition.

Raw EEG data are deposited on Open Science Framework.

We mention our exclusion criteria based on our pre-registration on Page 19, under *Methods/Subjects*. Subjects were excluded if they had more than 3 noisy channels in their EEG recording and/or they made the same choice in more than 90% of trials in our NATURAL condition.

We did not identify any outliers in the rest of our analyses.

The rest of the points above are not applicable to our study.

eLife Sciences Publications, Ltd is a limited liability non-profit non-stock corporation incorporated in the State of Delaware, USA, with company number 5030732, and is registered in the UK with company number FC030576 and branch number BR015634 at the address Westbrook Centre, Milton Road Cambridge CB4 1YG, UK | March 2019

Statistical reporting

- Statistical analysis methods should be described and justified
- Raw data should be presented in figures whenever informative to do so (typically when N per group is less than 10)
- For each experiment, you should identify the statistical tests used, exact values of N, definitions of center, methods of multiple test correction, and dispersion and precision measures (e.g., mean, median, SD, SEM, confidence intervals; and, for the major substantive results, a measure of effect size (e.g., Pearson's r, Cohen's d)
- Report exact p-values wherever possible alongside the summary statistics and 95% confidence intervals. These should be reported for all key questions and not only when the p-value is less than 0.05.

Please outline where this information can be found within the submission (e.g., sections or figure legends), or explain why this information doesn't apply to your submission:

We explain our statistical analysis methods in full detail on Pages 23-24 under Methods/statistical analysis.

We explain our criteria for multiple test correction on Page 24 under Methods/statistical analysis. We mention the statistical tests used, which mainly include paired t-tests and Pearson's r correlation, anytime we report the results of such tests in the Results section. Effect sizes are not included in the current version of the manuscript following the common practice, but they will be added if the paper is invited for revision.

The exact p-values are reported in the Results section and on Figures 3-5.

(For large datasets, or papers with a very large number of statistical tests, you may upload a single table file with tests, Ns, etc., with reference to sections in the manuscript.)

Group allocation

- Indicate how samples were allocated into experimental groups (in the case of clinical studies, please specify allocation to treatment method); if randomization was used, please also state if restricted randomization was applied
- Indicate if masking was used during group allocation, data collection and/or data analysis

Please outline where this information can be found within the submission (e.g., sections or figure legends), or explain why this information doesn't apply to your submission:

All participants completed a within-subject manipulation. Thus, group allocation procedures are not relevant here.

Additional data files ("source data")

- We encourage you to upload relevant additional data files, such as numerical data that are represented as a graph in a figure, or as a summary table
- Where provided, these should be in the most useful format, and they can be uploaded as "Source data" files linked to a main figure or table
- Include model definition files including the full list of parameters used
- Include code used for data analysis (e.g., R, MatLab)

- Avoid stating that data files are "available upon request"

Please indicate the figures or tables for which source data files have been provided:

All modeling and analysis MATLAB codes, including codes for making figures and reporting of all key statistics, will be made available after the manuscript is accepted for publication.
